# Improving Pelvic Floor Muscle Training with AI: A Novel Quality Assessment System for Pelvic Floor Dysfunction

**DOI:** 10.3390/s24216937

**Published:** 2024-10-29

**Authors:** Batoul El-Sayegh, Chantale Dumoulin, François Leduc-Primeau, Mohamad Sawan

**Affiliations:** 1 Department of Electrical Engineering, Polytechnique Montreal, Montreal, QC H3T 1J4, Canada; batoulelsayegh@gmail.com (B.E.-S.); francois.leduc-primeau@polymtl.ca (F.L.-P.); 2 Research Center, Institut Universtaire de Gériatrie de Montréal, Montreal, QC H3W 1W5, Canada; 3 School of Rehabilitation, Faculty of Medicine, Université de Montréal, Montreal, QC H3T 1J4, Canada; 4 CenBRAIN Neurotech, School of Engineering, Westlake University, Hangzhou 310024, China

**Keywords:** urinary incontinence, rehabilitation, pelvic floor muscle contraction, vaginal dynamometer, artificial intelligence

## Abstract

The first line of treatment for urinary incontinence is pelvic floor muscle (PFM) training, aimed at reducing leakage episodes by strengthening these muscles. However, many women struggle with performing correct PFM contractions or have misconceptions about their contractions. To address this issue, we present a novel PFM contraction quality assessment system. This system combines a PFM contraction detector with a maximal PFM contraction performance classifier. The contraction detector first identifies whether or not a PFM contraction was performed. Then, the contraction classifier autonomously quantifies the quality of maximal PFM contractions across different features, which are also combined into an overall rating. Both algorithms are based on artificial intelligence (AI) methods. The detector relies on a convolutional neural network, while the contraction classifier uses a custom feature extractor followed by a random forest classifier to predict the strength rating based on the modified Oxford scale. The AI algorithms were trained and tested using datasets measured by vaginal dynamometry, combined in some cases with digital assessment results from expert physiotherapists. The contraction detector was trained on one dataset and then tested on two datasets measured with different dynamometers, achieving 97% accuracy on the first dataset and 100% accuracy on the second. For the contraction performance classifier, the results demonstrate that important clinical features can be extracted automatically with an acceptable error. Furthermore, the contraction classifier is able to predict the strength rating within a ±1 scale point with 97% accuracy. These results demonstrate the system’s potential to enhance PFM training and rehabilitation by enabling women to monitor and improve their PFM contractions autonomously.

## 1. Introduction

Urinary incontinence (UI) is defined by the International Continence Society (ICS) as the complaint of involuntary loss of urine [1]. UI is common, affecting around 30% of women, with prevalence varying according to demographic factors like age and childbirth [2]. This condition reduces the patient’s quality of life and self-esteem, while also placing financial pressure on both patients and healthcare organizations [3,4].

Continence is multi-factorial but relies mainly on the urethral closure and detrusor control function. Pelvic floor muscle (PFM) integrity is of high importance in the closing of the urethra. When there is an increase in intra-abdominal pressure, a weak PFM will not be able to support and close the urethra, and thus a leakage will occur [1,5]. Pelvic floor muscle training (PFMT), the first-line treatment for UI, has been shown to improve urethral closure pressure and pelvic organ support, thereby preventing urinary leakage [5,6].

The ICS and the International Urogynecological Association (IUGA) recommend assessing the PFMs function prior to and during PFMT to teach effective PFM contraction and measure improvement, thereby giving the best training program for specific PFM dysfunctions [6]. According to the ICS, a normal PFM contraction involves “a constriction and inward (ventrocephalad) movement of the pelvic openings” [7]. The ability to perform a correct PFM contraction is a prerequisite for PFMT. However, as the PFM are invisible inside the pelvis, research indicates that many women lack the knowledge and ability to perform a correct PFM contraction [8,9,10]. Further, women reporting prior knowledge of PFMT may still perform PFM contractions incorrectly [11]. Studies have shown that the prevalence of women with PFM dysfunctions who cannot contract their PFMs correctly ranges from 12 to 55%, with this variation depending on differences in study design, participant characteristics, and assessment methods [12,13,14].

In 2014, a study conducted with 998 women showed that 70% were unable to voluntarily contract their PFMs correctly (ventrocephalad), without the feedback of a physiotherapist. Of those not able to perform a PFM contraction, 33.4% were personally convinced they were able to do it; 73.6% of participants showed an improvement in their PFM contraction after receiving verbal instructions [10]. Although verbal instruction has a positive effect, some women still need more than verbal instructions to correctly perform a PFM contraction [10,11,15].

In 2019, another study conducted on 82 women showed that only 33 of the women who participated in the study had an accurate self-perception of their own PFM contraction in relation to the categories of the Modified Oxford Scale (MOS) [8]. Therefore, for effective PFMT, it is essential that women have a proper understanding and confirmation of their ability to perform a correct PFM contraction.

Currently, there is no universal standard for assessing the correctness of PFM contractions in women. By far, the most common assessment method is digital palpation, which is used to assess, teach, and give feedback on the PFM contraction. However, this method relies on digital sensation by a physiotherapist and is subject to interpretation. A physiotherapist is expected to give instruction during different tasks, evaluate the task, and monitor the correctness of PFM contractions in participants. Beyond being a subjective measurement, this is not feasible, nor is it possible especially in group treatments, online rehabilitation, or during home PFM exercises. Therefore, there is a need for an objective and reliable PFM measurement tool to assess PFM function and to confirm the correctness of women’s PFMT techniques.

Various measurement methods have been developed for the evaluation and training of PFM [16,17]. Recently, PFM dynamometry has been suggested as a direct, reliable, and objective measurement tool [16,17,18]. However, presently, available dynamometers are limited to one functionality, either providing reliable measurements or offering feedback on the correctness of PFM contractions—but not both. Most marketed devices measure PFM force, yet fall short of guiding users through correct exercise contraction. Consequently, there is a need for a device that measures the PFM function and accurately detect PFM contractions [8].

Recognizing the importance of a real-time PFM contraction feedback assessment system in rehabilitation intervention, our objective is to design a PFM contraction quality assessment system. The latter is aimed at detecting PFM contraction in real-time as well as assigning a rating to the contraction based on different features and an overall rating. Different tasks were used to define PFM contraction and non-contraction. While the proposed quality assessment system has been designed, it is yet to be implemented in PFM function measurement tools, including a recent portable vaginal dynamometer built by our team. The newly achieved vaginal dynamometer enables the measurement of PFM forces in the standing position, which is the naturally occurring position for UI [17]. Once the PFM contraction quality assessment system is implemented, women will be able to use the portable dynamometer to train with direct feedback on PFM contraction, both at rehabilitation centers and at home.

The remaining parts of this paper include a summary of the main related prior-art publications in Section 2. Section 3 outlines the design of the novel PFM contraction quality assessment system. We present in Section 4 the experimental results of the system, which are subsequently discussed in Section 5. Finally, Section 6 provides the conclusions and outlines directions for future work.

## 2. Related Work

Based on our thorough review of the literature, and to the best of our knowledge, no previous work exists describing the implementation of a neural network model to differentiate between PFM contraction and non-contraction in the context of vaginal dynamometry. However, it should be noted that van den Noort et al. have utilized a convolutional neural network (CNN) for the assessment of PFM contractions using 4D ultrasound clips [19]. While CNNs have been applied to pelvic floor assessments using ultrasound, their application within vaginal dynamometry remains novel. Additionally, there is no previous work using AI algorithms to classify PFM strength based on digital palpation data. While several PFM dynamometers have been developed for research purposes, none of them incorporate the evaluation of PFM contraction. Notably, only two personal PFM dynamometers utilize hardware and/or an algorithm to differentiate between correct and non-PFM contraction [20,21].

The manufacturers of Pericoach, a commercially available personal PFM dynamometer, reported in a conference paper that the device was upgraded to detect and monitor exercise techniques using an algorithm that distinguishes specific movement patterns and scores them from good (1) to bad (−1) [20]. However, no definition is given for the specific movement patterns, and no information is available about the algorithm itself, the technique used for score development, the data used to train the algorithm and to create the technique scores, and whether or not validation was done for both the algorithm and technique scoring. Furthermore, the psychometric properties of this home trainer are not yet studied. More research is needed to assess the reliability and validity of measurements taken with Pericoach in different populations and conditions [16].

The manufacturers of Elvie, another personal PFM dynamometer available commercially, reported the ability of the trainer to specify the correct/incorrect contraction of a PFM maximum voluntary contraction (MVC). The device uses an accelerometer to measure the angular rotation of the device in situ, with the antero-cranial rotation (pitch) indicating the correctness of the PFM contraction. Theoretically, the cranial lift and anterior squeeze action of the PFMs during a correctly performed MVC generates positive antero-cranial rotation of the Elvie Trainer, while the caudal descent and relaxation of the posterior aspect of the PFMs during bearing down generate negative antero-cranial rotation. In a study performed in 2020 on 22 women, Elvie was able to correctly classify pelvic floor contractions and Valsalva maneuvers in supine and standing positions [21]. However, Elvie was tested solely on women who were already trained on PFM contraction. The accuracy of this classification should be tested on women who are naive to the task. Further, the Elvie Trainer had a poor correlation between the forces measured by the device and an intra-vaginal dynamometer, which may limit its ability to detect improvements in strength over time. As such, the device’s contractile force values and antero-cranial rotation values are not recommended as objective measures of PFM strength or for monitoring improvements in PFM strength with treatment. Further research is needed to evaluate whether or not the Elvie Trainer could be an effective home training tool to improve symptoms of pelvic floor dysfunction [21].

Currently, there exists no PFM dynamometer in both research and industry that offers a complete reliable system of measuring PFM function while also evaluating the PFM contractions. There is a lack of research on integrating systems that can detect and assess the quality of PFM contractions. To address these drawbacks, we propose a PFM contraction quality assessment system that detects PFM contractions and evaluates their quality across different features.

## 3. Materials and Methods

**Figure 1:**
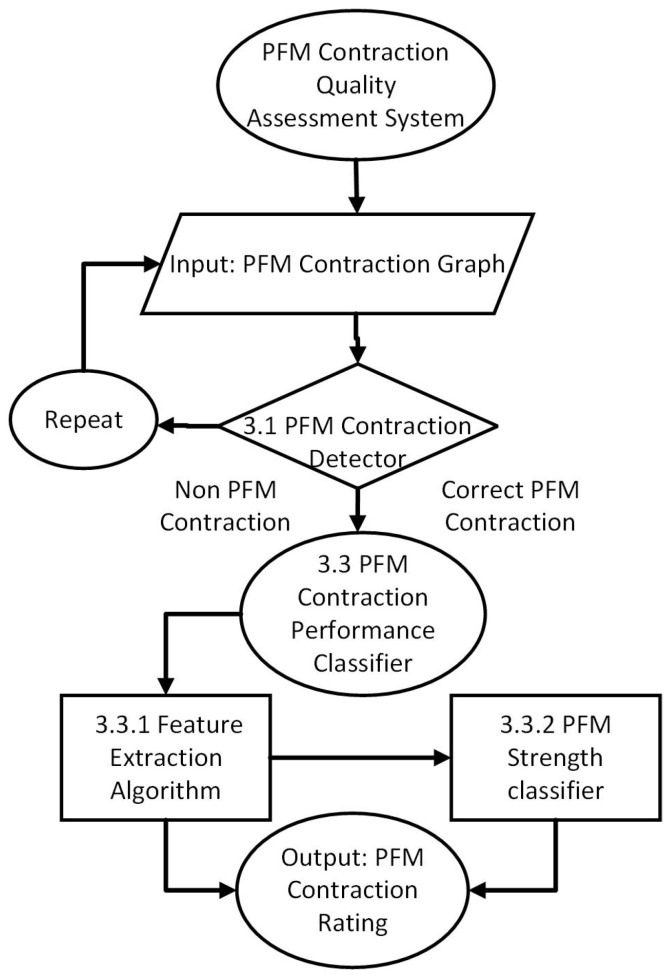
Overview of the quality assessment system implemented.

In this section, we describe the proposed quality assessment system, which is designed to differentiate between PFM contraction and non-contraction and quantify the quality of the maximal PFM contraction across different features (Figure 1).

Correct contraction of the PFM involves the precise activation of these muscles, which results in a constriction and upward (ventrocephalad) movement of the pelvic openings. Correct contraction can be quantified using a dynamometer by measuring a maximal voluntary contraction.

A non-PFM contraction is any action that deviates from the cranioventral movement, such as no movement or movement opposite to cranioventral. To define PFM contraction and non-contraction we used different tasks. During the rest task, there should be no movement of the PFM, making it a suitable proxy for a non-PFM contraction. In the pushing task, participants are asked to do the opposite of a contraction, usually resulting in minimal or no cranioventral movement, which is another proxy for non-PFM contraction.

For the cough task, since women may perform a pre-cough contraction known as the knack movement, only dynamometric recordings from participants who did not perform a knack during the digital palpation test preceding the dynamometric measurement in the same session were included. These tasks and assessments provide a comprehensive framework for differentiating between PFM contraction and non-contractions using a dynamometer.

### 3.1. PFM Contraction Detector

Over the past decade, deep learning has emerged as a transformative approach in automating the analysis of medical data, including time-series data like PFM force measurements [22]. By enabling the recognition of patterns in contraction data, deep-learning techniques, such as CNNs, have significantly enhanced the accuracy of classification, surpassing traditional methods. In this study, the PFM contraction detector is based on a CNN model, with key considerations focused on the quality of training data and the design of the model architecture.

#### 3.1.1. Training Dataset 1

To train and test the CNN model, PFM contraction and non-contraction measurements are needed. Dynamometric data were obtained from a previous study [23]. The study is a randomized non-inferiority trial that compared the effectiveness of group rehabilitation and individual physiotherapy in treating symptoms of stress/mixed urinary incontinence in community-dwelling older women. The trial was assessor-blinded, and 362 eligible participants were enrolled [23]; however, only 318 had dynamometric data. After receiving instructions on how to contract their PFM, participants underwent 12 weeks of PFM training, either individually or as part of a group of eight women. Baseline, post-treatment, and one-year follow-up measurements were taken using an intra-vaginal dynamometer [18,23]. It is important to note that participants were not naive to PFM contraction: they were required to demonstrate their ability to contract correctly to be eligible for participation [24].

Details on the participants’ inclusion/exclusion criteria are provided in the trial protocol [18], along with details of the study, which have been previously published [23,24]. The study protocol was approved by the research ethics board at both recruitment sites, and each volunteer provided written consent before participation (trial registration ClinicalTrials.gov Identifier: NCT02039830).

From the dynamometric data acquired from participants, we were interested in the following recording: (1) 5 s rest at a minimal dynamometer opening (11 mm) recorded twice, (2) 10 s maximal PFM contraction, repeated three times, and (3) three strong coughs in succession, repeated twice. The aforementioned data were extracted for the 318 participants. For some participants, there was less than what was required due to the unwillingness of the participants to complete the procedure and occasionally due to inadequately recorded data [23].

The data are time-series recordings of force (N) as a function of time. Graphs were examined visually by an expert with 6 years of experience in reading dynamometric data, to ensure the quality of the data. Any recording that did not include the corresponding task (rest, contraction, or cough) was excluded. For example, recordings of contractions or coughs that did not contain any movements were excluded. Graphs that were recorded partially (recording started late or recording ended early) were also excluded. Additionally, rest recordings that included any movement were also excluded.

#### 3.1.2. Signal Pre-Processing

Dataset 1 was recorded using an older version of the Montreal PFM dynamometer at a frequency of 1024 Hz [25]. For Dataset 2, recorded using a newly designed portable PFM dynamometer [17] and as a part of a recently developed exergame [26], a frequency of 20 Hz has been selected to meet the functional requirements of the exergame [26]. Considering that the future goal was to implement the CNN model in the newly designed PFM dynamometer [17], Dataset 1 has been down-sampled to a frequency of 20 Hz.

Prior to down-sampling, the triple cough recordings were partitioned such that each cough was represented as a separate graph. To down-sample Dataset 1 and ensure that the shape pattern remained unaffected, several steps were employed. First, a fourth-order Butterworth low-pass filter was used to remove any possible high-frequency components. The filter is designed with a cutoff frequency of half the new sampling rate, which is the maximum frequency that can be represented in the down-sampled signal according to the Nyquist–Shannon sampling theorem. Then, using the decimation method, the filtered data were down-sampled to 20 Hz, which is represented by:

(1) $$y\left[n\right]=x_{\mathrm{filtered}}\left[nM\right]$$

where y[n] is the down-sampled signal and $x_{\mathrm{filtered}}\left[nM\right]$ is the filtered signal. The down-sampled signal is obtained by selecting every *M*th sample of the filtered signal. To be able to compare the original and down-sampled signals using the same support (the set of x-values for which a discrete signal f(x) is defined), the original signal is then reconstructed from the down-sampled signal based on the zero-order hold method. A fourth-order Butterworth low-pass filter is then used to smooth the reconstructed signal curve.

To show the difference between the original and the reconstructed signal, root mean squared percentage error (RMSPE) has been calculated based on the following formula [27]:

(2) $$\mathrm{RMSPE}=\sqrt{\frac{1}{n}\sum_{i=1}^{n}{\left(\frac{y_{i}-y_{p}}{y_{i}}\right)}^{2}}\times 100,$$

where $y_{i}$ is the measured value at time t for the original signal and $y_{p}$ is the predicted value at time t for the reconstructed signal. Additionally, the top-three dominant frequencies were calculated for both the original and down-sampled signals to compare the signal frequency spectrum. The RMSPE was also used to compare the dominant frequencies of the original signal and the down-sampled signal.

#### 3.1.3. CNN Architecture and Testing Setting

**Figure 2:**
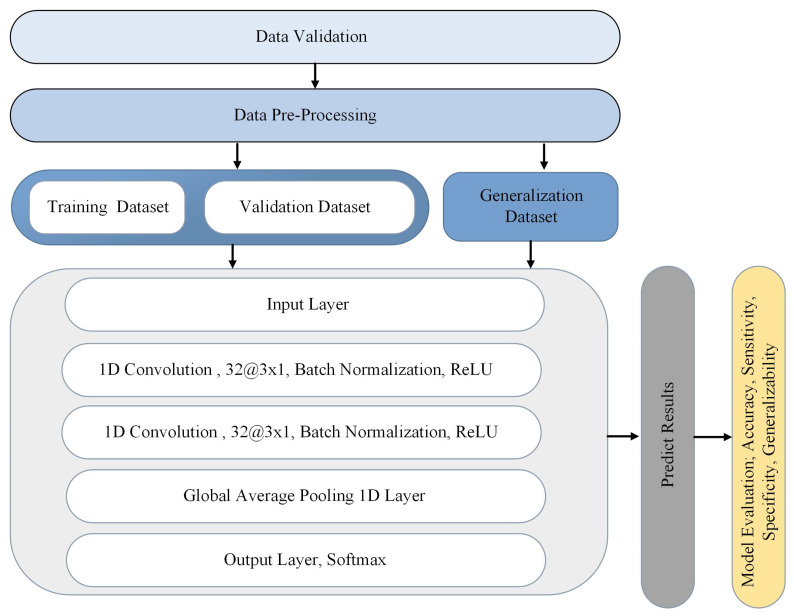
Overview of CNN model architecture and data processing pipeline.

The overall CNN architecture is shown in Figure 2. The proposed CNN model is a one-dimensional stacked CNN (1DSCNN) model. This CNN model is composed of a 1D input layer, two convolutional layers (each with 32 filters and a kernel size of 3), and a global average pooling layer. Batch normalization is applied after each convolutional layer to adjust the activations of the previous layer across the batch. Rectified linear unit (ReLU) activation functions are used to introduce non-linearity in the network after each batch normalization layer. Global average pooling is applied after the second convolutional layer to convert the feature maps into a single feature vector by taking the average of all feature maps across each channel. This reduces the dimensionality of the feature representation. A dense layer with a softmax activation function is used as the output layer to map the feature vector to the class probabilities. The number of neurons in the output layer is equal to the number of classes in the classification task.

The model is trained with the Adam optimizer, sparse categorical cross-entropy loss function, and sparse categorical accuracy metric. The model is trained for 100 epochs with a batch size of 32. The training is stopped early if the validation loss does not improve for 50 epochs. The learning rate is reduced by a factor of 0.5 if the validation loss does not improve for 20 epochs. Finally, the model is evaluated on a test set, and its accuracy, loss, specificity, and sensitivity are reported.

The implementation was carried out using Keras 2.12 on a Dell Inspiron 7559 laptop (Dell, Round Rock, TX, USA), equipped with an NVIDIA GeForce GTX 960 GPU (NVIDIA, Santa Clara, CA, USA). We used Python 3.10.7 as the programming language, with Visual Studio Code (VSCode 1.94.2) as the development environment.

### 3.2. Validation of the CNN Model

The validation of the CNN model was done in two steps. The first step was to validate the model with data from the original training data series. This step was intended to validate that the model works successfully within the same device. The second step was to validate the model across a different dataset from a different device, which will allow testing the generalizability of the model, that is the ability of the model to perform effectively with new data.

#### 3.2.1. Validation of the CNN Model on Dataset 1

To estimate the performance, five-fold cross-validation was used. Five-fold cross-validation is a common cross-validation technique in machine learning. It splits a dataset into five subsets or “folds” of roughly equal size. During the cross-validation process, one fold is used as the test set while the remaining four folds are utilized for training. This process is repeated five times, ensuring that each fold of the data is tested once and remains unseen during training. During each run, the model’s performance metrics (accuracy, sensitivity, specificity) are recorded. Then, the average accuracy, sensitivity, and specificity across all five runs is calculated to obtain a robust estimate of the model’s performance.

#### 3.2.2. Generalization Testing on Dataset 2

To test the generalization of our model, we used Dataset 2, which was obtained from a feasibility study. This study is a clinical trial that compares the effectiveness of individualized video game training (VITAAL Exergame) for older adults with mobility impairments and/or urinary incontinence [28]. A total of 38 eligible participants were enrolled; however, only 13 had dynamometric data. Participants were randomized into a 12-week stepping exergame training group or a traditional exercise (active control) group. Baseline and post-treatment measurements were taken using the newly developed portable PFM dynamometer [17].

Eligible participants were incontinent community-dwelling women, aged 60 and over with symptoms of mixed UI or urge UI, who reported at least three episodes of urine loss per week during the preceding three months. Mixed/urge UI was confirmed by the Questionnaire for Urinary Incontinence Diagnosis. Exclusion criteria included body mass index ≥40, important pelvic organ prolapse (POPQ > 2), physiotherapy treatment or surgery for UI or pelvic organ prolapse in the past year, or any medication and co-morbidities/risk factors interfering with the study. The study protocol was approved by the research ethics board at both recruitment sites, and each volunteer provided written consent before participation (trial registration ClinicalTrials.gov Identifier: NCT04587895).

From the data acquired from participants on the baseline and at post-treatment, we were interested in the following recording: (1) rest recorded twice, (2) maximal PFM contraction, repeated twice, (3) three strong coughs in succession, repeated twice, and (4) a push, if possible. The data were extracted for the 13 participants. For some participants, there was no data or less data than what was required due to the unwillingness of the participants to join/complete the procedure, malfunctioning of the phone application, and occasionally inadequately recorded data. The data are time-series recordings of force (N) as a function of time. Graphs were examined visually to ensure the quality of the data. Any recording that did not include the corresponding task (baseline, contraction, cough, or push) was excluded.

Table 1 provides a summary of the final datasets utilized for the CNN model following exclusions.

### 3.3. Maximal PFM Contraction Performance Classifier

We propose a novel algorithm to autonomously quantify the PFM function in women. Our algorithm takes PFM contraction graphs as input and outputs a rating across different features. This is accomplished by extracting specific features from the PFM contraction graphs and evaluating them. The PFM contraction graphs are recorded during the maximal voluntary contraction task, following a cue to contract and maintain the contraction for 10 s [18,23].

Table 2 presents the definition of each feature used to evaluate the different aspects of PFM contraction. Hence, this study aims to introduce an autonomous algorithm capable of extracting and evaluating PFM contractions through the identification and evaluation of these specific features.

**Figure 3:**
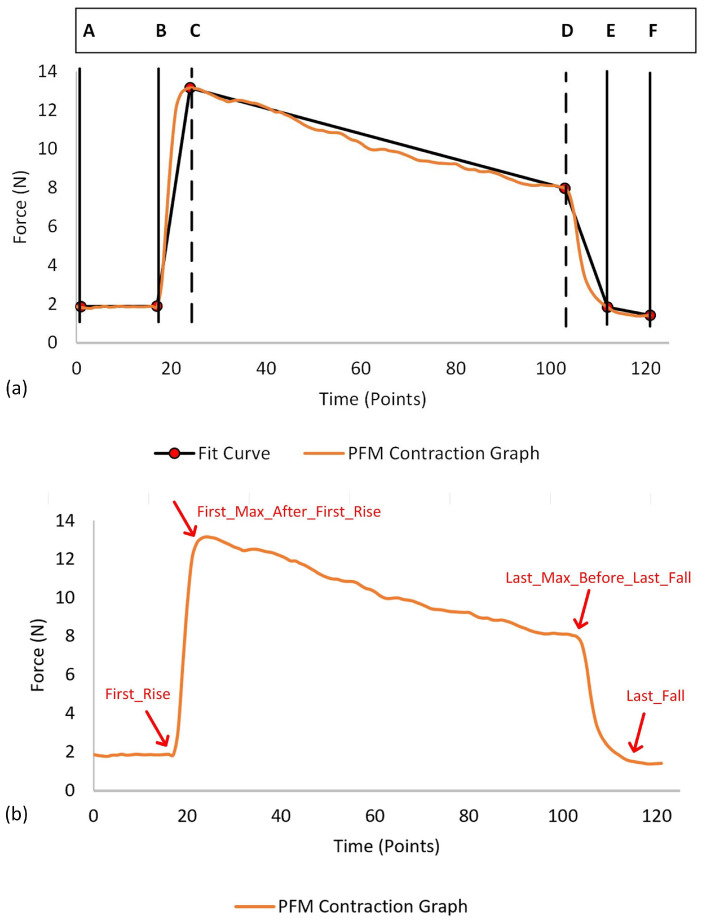
(**a**) Example of the trapezoid model fitted to a contraction graph. (**b**) Feature extraction algorithm key aspects.

To extract the required features from a PFM contraction graph, we developed a mathematical piecewise linear model. This model approximates the typical trapezoidal shape of the contraction graph by dividing it into five distinct linear segments. Figure 3 illustrates an example of fitting a PFM contraction graph into this trapezoidal model. The key parameters of the model are the time values at points B, C, D, and E, which define the critical phases of the contraction. The model autonomously identifies these time values to accurately capture the essential features of the contraction. The trapezoid model can be described by the following equation:

(3) $$F_{c}\left(x\right)=\left\{\begin{array}{cc} m_{i}t+b_{i} & A\leq t<B,\\ m_{r}t+b_{r} & B\leq t<C,\\ m_{c}t+b_{c} & C\leq t<D,\\ m_{f}t+b_{f} & D\leq t\leq E. \end{array}\right.$$

Note that the line parameters and the time values A to E depend on each other, such that the model has in total 8 degrees of freedom that must be identified.

Figure 3 shows the key aspects of the feature extraction algorithm. The vertices of the contraction are autonomously extracted based on the following steps. First, each signal’s baseline mean value is subtracted from the corresponding contraction signal. Then, the first derivative of each signal is calculated. Next, to detect the first rise (refer to Figure 3), we created a function called “Detect First Rise”. The first rise is defined as the point where the contraction starts and is characterized by the point where a drastic increase starts (point B in Figure 3). The “Detect First Rise” function uses the threshold-based method, taking the derivative data array and a list of threshold values as arguments. Multiple thresholds are required because a single threshold cannot account for the substantial variations in y-values across different datasets: this ensures correct detection of point B in diverse data. The function loops through the derivative values and finds the indices where the derivative data are greater than the first threshold. If none is found, the function repeats the step taking the second threshold, and so on until the function returns the index of the first point where the derivative is higher than the given threshold.

**Figure 4:**
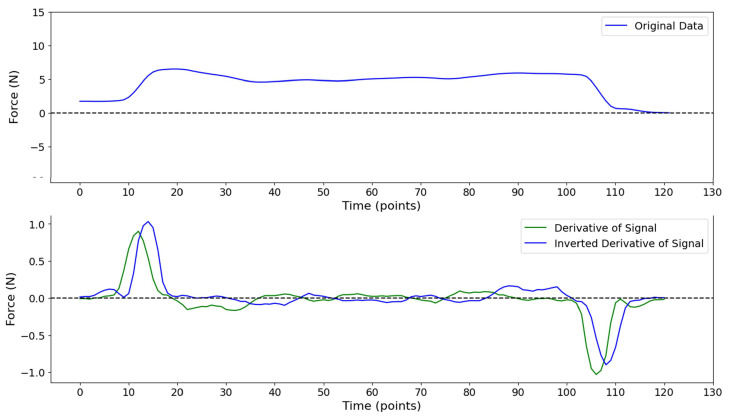
Example of original data derivative and inverted derivative.

To detect the last fall (refer to Figure 3), we developed a function called “Detect Last Fall”. The last fall is defined as the point where the contraction ends, marked by a significant decrease in the signal (point E in Figure 3). Specifically, a significant decrease is identified when the derivative of the force signal falls below a specified threshold, indicating that the force is decreasing at a rate that suggests the end of the contraction. Similar to the detection of the first rise, the “Detect Last Fall” function utilizes a threshold-based method. It takes a derivative data array and a list of threshold values as inputs. To transform the last fall point into a first rise scenario, the y-values of the derivative signals are reversed and mirrored with respect to the x-axis. This inversion effectively turns the detection of the last fall into the detection of a first rise, simplifying the process. Figure 4 illustrates an example of a PFM contraction signal along with its corresponding first derivative and inverted first derivative curves. The function then iterates through the list of threshold values, searching for indices where the derivative data exceeds the current threshold. Once the function identifies the first point where the derivative surpasses the threshold, it returns this index as the last fall point. The actual index of the last fall is then calculated by subtracting the obtained index from the total number of points, effectively mapping the detected point back to its original position in the signal.

After identifying the start and end of the contraction (first rise and last fall), the algorithm detects the first maximum after the first rise and the last maximum before the last fall using the two created functions “Detect First Max After First rise” and “Detect Last Max Before Last Fall”, respectively. These functions first calculate the zero-crossings of the derivative data, which are the points where the derivative changes sign (a zero derivative is a derivative of a maximum or a minimum). Then, the “Detect First Max After First Rise” function finds the index of the first maximum point after the first rising point (point C in Figure 3). Similarly, the “Detect Last Max After Last Fall” function finds the index of the last maximum point before the last falling point (point D in Figure 3).

Once the coordinates of the key parameters (B, C, D, and E) are determined, the trapezoid equation is computed and a mathematical fit is created for each signal, as described in Equation (3). Using this equation, the algorithm calculates various features essential for evaluating different PFM training tasks. The following are the features along with their respective computation methods:

Rising Slope: defined as the slope between points B and C and is calculated according to the following equation:

(4) $$m_{r}=\frac{F_{C}-F_{B}}{t_{C}-t_{B}},$$

where $F_{C}$ is the force value at C, $F_{B}$ is the force value at B, $t_{C}$ is the x index at C, and $t_{B}$ is the x index at B.

Falling Slope: defined as the slope between points E and D and is calculated according to the following equation:

(5) $$m_{f}=\frac{F_{E}-F_{D}}{t_{E}-t_{D}},$$

where $F_{D}$ is the force value at D, $F_{E}$ is the force value at E, $t_{D}$ is the x index at D, and $t_{E}$ is the x index at E.

Area Under the Contraction Curve: The area under the contraction curve of the piecewise linear model signal, between points B and E, is calculated using the following trapezoidal rule equation:

(6) $$\int_{a}^{b}f\left(t\right)dt\approx \frac{2}{w}\left[f\left(a\right)+2\sum_{i=1}^{n-1}f\left(t_{i}\right)+f\left(b\right)\right],$$

where a and b are the t indices of the first rise and last fall, respectively, n is the number of subintervals (trapezoids) used in the approximation, $t_{1}$, $t_{2}$, …, $t_{n-1}$ are the equally spaced points within the interval [a, b] where the function f(t) is evaluated, and w is the width of each trapezoid, given by:

(7) $$h=\frac{b-a}{n},$$

The limits a and b are flexible and can be adjusted based on the instructions given for the task.

Global Maximum: The $\Delta F_{\max}$ is defined as the global maximum force of each signal and is calculated by identifying the maximum force value:

(8) $$F_{c}\left(t\right)\leq \Delta F_{\max}\;;\;A\leq t\leq E,$$

The Average Force: The average force is defined as the mean of forces between points B and E and is calculated as follows:

(9) $$\bar{F_{c}}=\frac{1}{T_{E}-T_{B}}\sum_{i=B}^{E}F_{c},$$

The Contraction Time: The contraction time is defined as the difference between the point E index and point B index, which is dependent on the instruction given during the task:

(10) $$T_{c}=t_{E}-t_{B},$$

Muscle Relaxation: The $\Delta F_{f-i}$ is defined as the baseline force average:

(11) $$\Delta F_{f-i}={\bar{F}}_{AB},$$

Finally, the features of each signal are normalized to within the range of 0 to 1 and saved as a row in an Excel file along with the ratings of each feature.

Further, we introduced an overall rating scale to assess PFM contraction performance strategy regardless of the task. This scale provides a comprehensive assessment of the PFM contraction and takes into account all relevant features. The proposed overall rating is calculated based on the following formula:

(12) $$Overall\;rating\;\left(\%\right)=\left(c_{1}\cdot \Delta F_{max}+c_{2}\cdot T_{c}+c_{3}\cdot \bar{F_{c}}+c_{4}\cdot \frac{\left(mr+mf\right)}{2}+c_{5}\cdot \Delta F_{f-i}\right)\times 100,$$

where $c_{1}$, $c_{2}$, $c_{3}$, $c_{4}$, and $c_{5}$ are the weights of each feature contributing to the overall rating.

The features and weights included in the overall rating scale are flexible and not fixed. They are based on clinical input, specifically the cues clinicians provide. The current formula serves as an initial framework for evaluating PFM contractions. It is intended to be refined over time as more clinical data and feedback become available.

#### PFM Strength Classifier

A data-driven approach was adopted to predict the classification of the maximum PFM contraction graphs based on MOS. The MOS is a grading system used to evaluate the strength of PFM during a digital palpation exam, with six different grades ranging from 0 to 5 based on the level of contraction strength, as follows [30]:

0 = nil;
1 = flicker;
2 = weak;
3 = moderate;
4 = good;
5 = strong

Maximum PFM contraction graphs from Dataset 1 were used in the development of the classification model. A digital palpation examination of the 318 participating individuals was performed during pre- and post-evaluations by six expert physiotherapists. Data labeling was done by correlating the digital-palpation-labeled MOS with the PFM contraction graphs.

Given the subjectivity inherent in digital palpation, as described in the relevant literature [31], we accounted for variability in the scoring between different clinicians by allowing a margin of ±1 MOS class. This adjustment addresses the expected variability and provides a more realistic representation of model performance.

**Figure 5:**
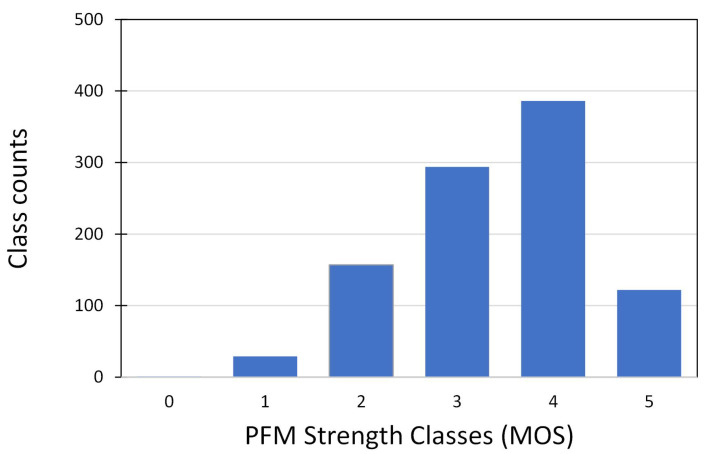
Bar chart of the class distribution of PFM strength recordings from Dataset 1.

The input data consisted of the features extracted normalized to within the range of 0 to 1. The output labels are the different MOS classes. Figure 5 shows the class counts for each MOS class used in the classification process. Since this dataset represents real MOS grading from a previous study with no control over PFM strength variations, class imbalances are expected. For example, as can be seen in Figure 5, there are more participants with an MOS grade of 3 than participants with an MOS grade of 0. Due to a lack of sufficient data for MOS grade 0 (1 participant out of 318), this class has been excluded from the classification process.

A combination of decision tree-based classification models, a statistical model, and a tree-based ensemble learning model were used. These models are well-established and widely used for similar classification tasks involving signal data. A random forest classifier (RFC) and a TOP random forest classifier (TRFC) have been used to classify the input data. The RFC and TRFC models work by creating multiple decision trees on randomly selected subsets of the data and then aggregating the results of these trees to make the final classification. The only difference is that the TRFC model incorporates an extra feature selection step by selecting a subset of features based on their importance scores, which are computed by the RFC algorithm during the training phase.

Extreme gradient boosting (XGB), implementing a gradient boosting framework, was also used to classify the aforementioned data. The XGBoost technique uses iterative training to sequentially train a set of weak prediction models, often decision trees. Every new model aims to correct the flaws of the prior models to produce a more precise forecast model.

Finally, a logistic regression (LR) classification model has also been used. The XGB works by fitting a logistic function to the input data, which converts the input variables to the output likelihood of the observation falling into a specific class.

Data were split randomly into a training set (80% of total data) and a testing set (20% of total data). The class imbalance was maintained to reflect the original distribution of the dataset. Each model has been trained on the labeled data. Once trained, the models were used to predict the class of the testing set. The performance of each model was evaluated and compared, taking into consideration the ±1 MOS class adjustment to align with the inherent variability in clinical assessments, as mentioned earlier.

## 4. Results

### 4.1. CNN Model Performance Evaluation

In this section, we evaluate the performance of our CNN model in classifying PFM contraction graphs. We present the results in terms of accuracy, sensitivity, and specificity, demonstrating the effectiveness of the model in distinguishing between PFM contractions and non-contractions.

#### 4.1.1. Down-Sampling Dataset 1 Evaluation

**Figure 6:**
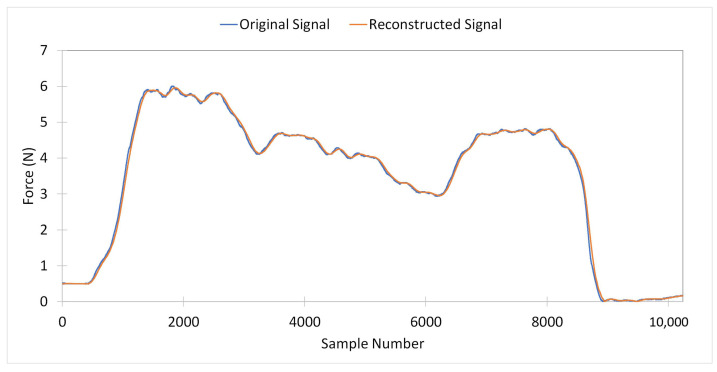
Original signal versus reconstructed signal of one PFM contraction from Dataset 1.

Figure 6 displays an original PFM contraction signal from Dataset 1 alongside its corresponding reconstructed signal. The average RMSPE calculated for Dataset 1 between original and reconstructed signals has been computed to be 2.58%.

**Figure 7:**
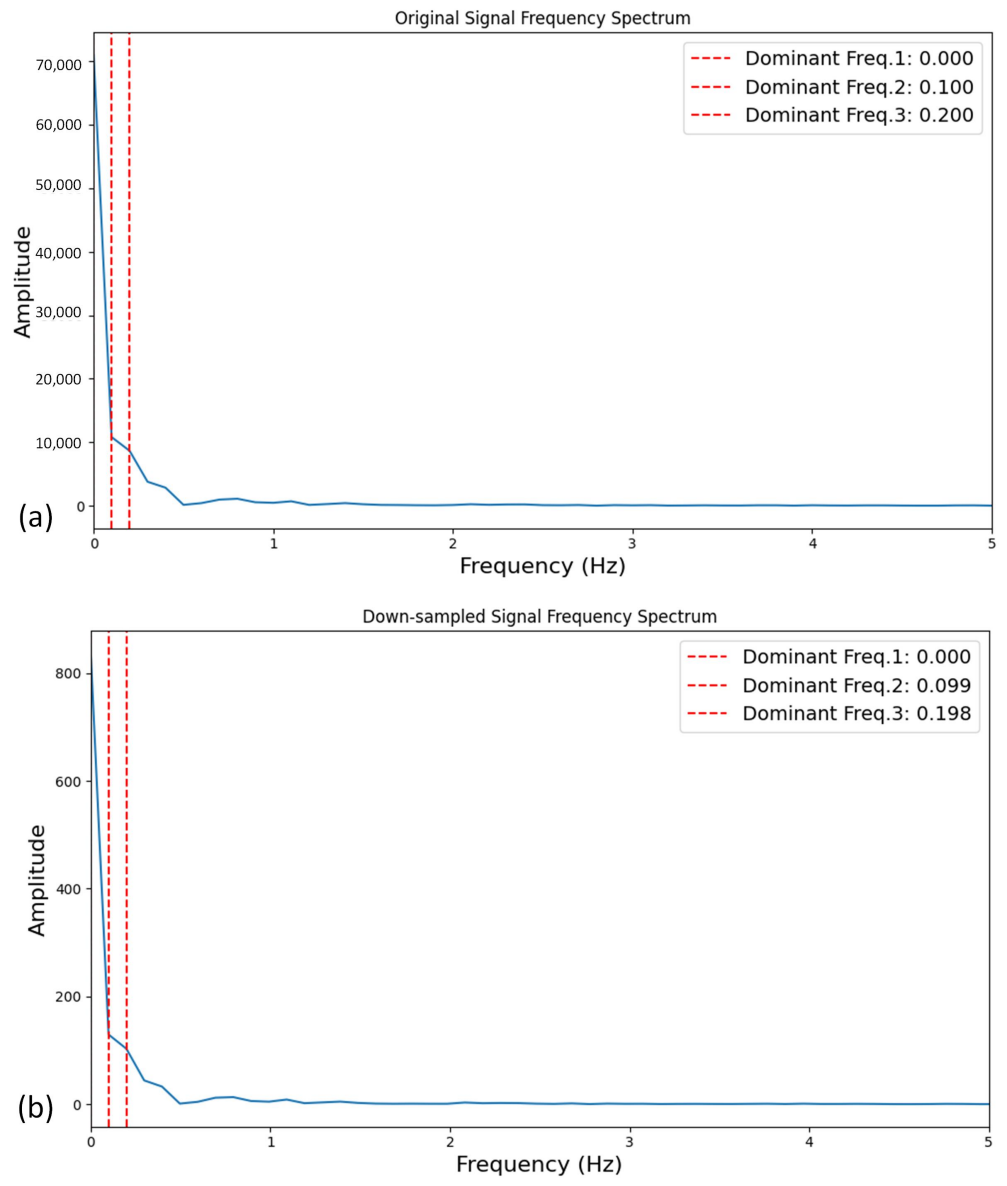
Top-three dominant frequencies for (**a**) original signal versus (**b**) down-sampled signal for one PFM contraction from Dataset 1.

Furthermore, Figure 7 illustrates an example from Dataset 1 of the frequency spectrum with the top-three dominant frequencies for an original signal and its corresponding down-sampled signal. The average RMSPE for the top-three dominant frequencies between the original and down-sampled signals was computed to be 0.65% for Dataset 1.

#### 4.1.2. Validation on the Dataset 1

**Figure 8:**
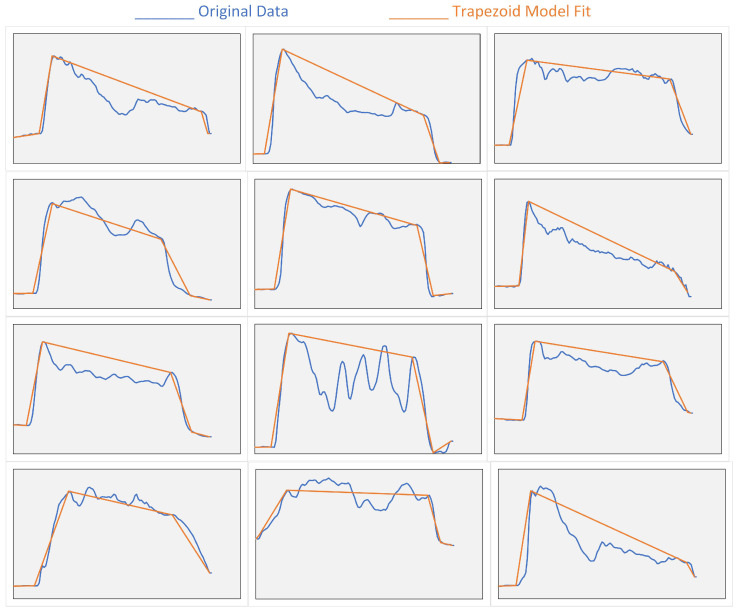
Examples of PFM contraction signals and their corresponding trapezoid model fit.

The CNN model performance has been evaluated on Dataset 1 by computing the model accuracy, sensitivity, and specificity. Figure 8 shows examples of PFM contraction signals and their corresponding trapezoid model fit.

**Figure 9:**
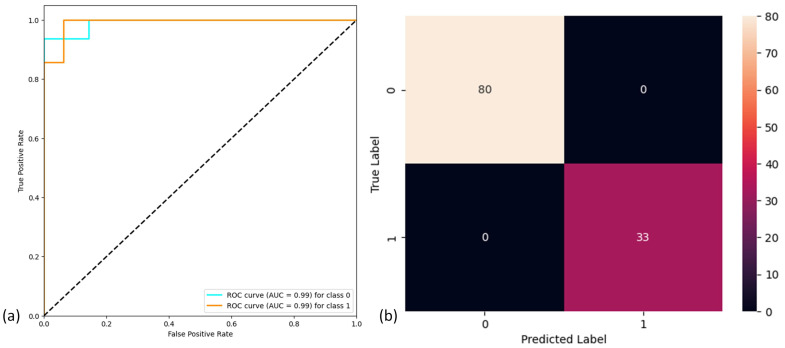
(**a**) Receiver operating characteristic curve for the CNN model. (**b**) Confusion matrix for CNN model for Dataset 2.

The model performance evaluation based on five-fold cross-validation is shown in Table 3. The model achieves high accuracy (96.66%), high sensitivity (96.66%), and high specificity (93.75%). Figure 9 shows the receiver operating characteristic curve (ROC) for the CNN model along with the area under the curve (AUC) values for each class. The model exhibits excellent AUC for both classes (99%).

#### 4.1.3. Generalization Testing on Dataset 2

The generalization ability of our CNN model was evaluated using Dataset 2. We directly applied our CNN model to Dataset 2 without training the model on it. Similarly, the model performance was evaluated in terms of accuracy, specificity, and sensitivity.

Figure 9 shows the confusion matrix obtained from Dataset 2 using the CNN model implemented and tested with Dataset 1. Table 3 shows that the model exhibits high accuracy (100%), high sensitivity (100%), and high specificity (100%).

### 4.2. Quality Algorithm

#### 4.2.1. Validation of the PFM Strength Classification

The classification of PFM contractions into the different MOS classes has been evaluated across the four different statistical classification methods. To evaluate the performance of each method, accuracy (A), precision (P), recall (R), and F1 score (F1) have been computed based on the following formulas [32]:

(13) $$Ac=\frac{TPc+TNc}{TPc+TNc+FPc+FNc},$$

(14) $$Pc=\frac{TPc}{TPc+FPc},$$

(15) $$Rc=\frac{TPc}{TPc+FNc},$$

(16) $$F1c=\frac{2\times Pc\times Rc}{Pc+Rc},$$

where $TP_{c}$ represents the number of data from class c identified as class c, $TN_{c}$ represents the number of data not from class c identified as a different class from class c, $FP_{c}$ are data that are not from class c identified as class c, and $FN_{c}$ are data from class c identified as a different class from class c.

The quality of the overall classification is assessed in two ways: macro averaging and weighted averaging. Macro averaging computes the measured average, regardless of the class distribution. The weighted average takes into account class imbalances by computing the average with respect to the class counts in the training set as follows:

(17) $$\begin{array}{cccccc} Pm & =\frac{1}{c}\sum_{c=1}^{c}P_{c}, & \;Rm & =\frac{1}{c}\sum_{c=1}^{c}R_{c}, & \;F1m & =\frac{2\times Pm\times Rm}{Pm+Rm} \end{array}$$

(18) $$\begin{array}{cccccc} Pw & =\frac{\sum_{c=1}^{c}TP_{c}}{\sum_{c=1}^{c}\left(TP_{c}+FP_{c}\right)}, & \;Rw & =\frac{\sum_{c=1}^{c}TP_{c}}{\sum_{c=1}^{c}\left(TP_{c}+FN_{c}\right)}, & \;F1w & =\frac{2\times Pw\times Rw}{Pw+Rw} \end{array}$$

where m indexes macro averaging and w indexes weighted averaging.

Table 4 presents the overall accuracy, precision, recall, and F1 score for the four classification methods within ±1 of each MOS class. The RFC classification model achieved the highest accuracy (95.56%), precision (89%), recall (88%), and F1 score (88%), outperforming the other methods, including XGB, LR, and TRFC. Therefore, the RFC classification model was considered the most effective approach for this particular classification task.

Table 5 shows the RFC method in-class scores for the precision, recall, and F1 score within ±1 of each MOS class. The RFC method exhibits medium to high precision scores (from 77 to 100%), medium to high recall scores (from 62 to 100%), and high F1-scores (from 76 to 100%) on each activity class. The overall weighted average precision, recall, and F1-score across all activities are 89%, 88%, and 88%, respectively.

Further review of the data where the RFC method’s predictions did not match the MOS revealed that 23% of these mismatches were due to participants moving their buttocks when they were supposed to only contract their pelvic floor muscles. The recording of buttock movement was performed during the evaluation sessions, where the physiotherapist visually observed and documented any such movements. After removing these particular cases and analyzing the data again, the accuracy increased to 96.53%, the overall weighted average precision remained at 89%, but the recall improved to 93%, and the F1 score went up to 90%.

#### 4.2.2. Algorithm Result

In our efforts to analyze PFM contraction data, we first used a code designed to fit the data to a trapezoidal function. This approach aimed to minimize the difference between the function and the actual data by adjusting parameters while adhering to constraints on the slopes of the rising and falling edges. Despite the theoretical robustness of this method, our rule-based feature extraction algorithm proved to be more effective. The superior performance of the rule-based algorithm can be attributed to its ability to incorporate specific clinical constraints—key parameters of the model—into the feature extraction process. These constraints include the points B, C, D, and E, which define the critical phases of the maximal pelvic floor contraction. By ensuring that the extracted features align with these clinically relevant parameters, the rule-based algorithm enhances the practical utility of the analysis in real-world clinical settings.

The algorithm’s autonomous ability to extract the features specified in Table 2 from PFM contraction graphs was evaluated using a set of PFM MVC graphs from Dataset 1. The dataset used to verify the algorithm consisted of 900 PFM MVC graphs from 318 participants. First, features were extracted autonomously using the algorithm. Then, the four corners of each MVC were manually identified, and the features’ values were re-calculated. The features’ values obtained from the algorithm were then compared to the manually extracted values.

The comparison between the features’ values obtained from the algorithm and the manually extracted values was done by calculating the mean absolute error (MAE) and standard deviation of the absolute forecasting error, $e_{i}$, based on the following formulas:

(19) $$e_{i}=y_{i}-{\hat{y}}_{p},$$

where $y_{i}$ is the manually calculated value and $y_{p}$ is the value calculated by the algorithm for the PFM contraction signal.

(20) $$\mathrm{MAE}=\frac{1}{n}\sum_{i=1}^{n}\left|e_{i}\right|,$$

The RMSPE for each feature was also calculated based on Equation (2). The RMSPE values obtained were then compared to a predetermined threshold (5%). The RMSPE values obtained were less than the threshold, indicating that the features were correctly extracted (Table 6).

### 4.3. PFM Quality Assessment System Comparison to State-of-the-Art

Table 7 presents a comparison of the method used and results achieved in this study with the state-of-the-art. The table shows that the proposed novel quality assessment system is the first to incorporate a CNN algorithm for the detection of PFM contraction and an autonomous maximal PFM contraction performance classifier. The obtained results indicate that the proposed quality assessment system outperforms the other state-of-the-art methods/devices in terms of accuracy, sensitivity, and specificity.

## 5. Discussion

We accomplished a novel quality assessment system for the classification and quantification of maximal PFM contraction in women. To the best of our knowledge, this is the first reported quality assessment system of this kind.

This paper presents three significant contributions. Firstly, we introduce a CNN-based PFM contraction detector specifically designed for the classification of PFM contractions. To the best of our knowledge, this is the first application of a CNN model for this purpose. Secondly, we provide an automated method for the evaluation of PFM strength based on MOS. Lastly, we propose a universal algorithm for autonomously rating the quality of maximal PFM contractions across different features. These contributions demonstrate the potential of our system to improve the accuracy and efficiency as well as to facilitate the clinical evaluation of PFM function.

The CNN model implemented for PFM contraction detection achieves an average accuracy of 97%, indicating its potential for accurately distinguishing between PFM contraction and non-contraction. After validating our CNN model through an independent dataset obtained from a newly developed PFM dynamometer, we observed a generalizability accuracy of 100%. This indicates that the model has been effectively trained to distinguish between PFM contraction and non-contraction. Moreover, further evaluation is necessary to determine the model’s robustness in more diverse and less-controlled environments. To address this, future studies should focus on evaluating the model’s performance on a more variable dataset, including data that has not been extensively cleaned, as well as investigating the effectiveness of signal feature extraction. These studies will help us assess the model’s clinical applicability and ensure that it generalizes well beyond the current dataset. The results obtained in this study represent a significant step in the development of an accurate and reliable method for assessing pelvic muscle function. This achievement is particularly noteworthy given the importance of performing correct PFM contraction during PFMT for pelvic floor disorder management such as UI.

The CNN model designed in this study has the potential to be integrated into different PFM assessment tools that can assist both patients and clinicians in accurately providing feedback on PFMT contraction. Regardless of the type of device used to record the PFM contractions, it is expected that this model could effectively detect PFM contractions. Additional research is necessary to determine the applicability of the accomplished CNN model to different PFM strength-measuring devices with different contraction tasks, such as endurance, rapid contractions, and muscle relaxation.

The RFC classifier based on digital palpation exhibited a high classification accuracy of 96.53% for classifying PFM contractions within the ±1 MOS class, which is notable given the subjective nature of digital palpation. A within ±1 MOS class classification was deemed acceptable due to the inherent discrepancies in the MOS grading between clinicians. It should be noted that inconsistencies between the RFC classification and the MOS were observed in some cases: for example, a high MOS accompanied by a low PFM contraction strength or a low MOS score accompanied by a high PFM contraction strength. Based on the physiotherapists’ observations during the study, in the cases with high discrepancies, a high MOS accompanied by a low PFM contraction strength could be caused by the patient’s discomfort or pain during the examination, resulting in incomplete contraction. Conversely, a low MOS score accompanied by a high PFM contraction strength could be attributed to compensatory movements such as buttock lifting during the examination.

These findings suggest that the subjective nature of digital palpation remains a challenge for accurate PFM contraction assessment and highlight the potential of the implemented RFC algorithms in improving the reliability of the assessment. One of the limitations of this study is that all participants in Dataset 1 were able to contract their PFMs, leading to a skew in the data toward an MOS score of 3 or higher. Further studies should therefore be conducted on a larger population in patients with a wide range of PFM strengths and MOS classifications from 0 to 5. In addition, effort should be made to choose patients with no prior PFM pain to reduce bias. Moreover, this study represents an important step towards developing a reliable and objective method for assessing PFM contraction strength based on MOS.

In this study, a maximal PFM contraction performance classifier was designed to rate various features of a PFM contraction and provide direct autonomous feedback. Currently, PFM contraction is evaluated through digital palpation tasks or post-evaluation of recorded tasks using an assessment tool. However, digital palpation tasks are subjective and lack a global reference for comparison, while post-evaluation is time-consuming. The performance classifier results showed that the classifier was able to correctly extract the specific features of the PFM contraction graphs, with RMSPE values below a threshold of 5% for each feature. Therefore, it can be concluded that the classifier has the autonomous ability to extract specific features from PFM maximum contraction graphs accurately.

One of the key strengths of the maximal PFM contraction performance classifier accomplished in this study is its ability to provide sub-ratings for different underlying features. This allows for a more detailed analysis of maximal PFM contractions and identification of areas of weakness in participants, which can be used to provide the best training program for specific PFM dysfunctions. However, the importance of specific features depends on the evaluator’s instructions given during the task. While our classifier has effectively identified a range of features for analyzing maximum voluntary contraction during the strength task, these features can also be applied to other tasks, such as endurance, muscle relaxation, or rapid contractions. By adjusting the evaluator’s instructions, we can determine which features are most relevant for each type of assessment, allowing for a tailored analysis of different tasks.

To advance standardization and simplify the evaluation of PFM contraction, we developed an overall rating scale integrating multiple features into a unified metric. This scale aims to provide a comprehensive assessment by incorporating all relevant features of PFM contraction. However, the successful implementation of this scale necessitates ongoing collaboration between clinicians and engineers to ensure that the features and instructions are well-coordinated. While the initial formula provides a foundational example, it is expected to evolve, potentially incorporating additional features or adjusting feature weights as more clinical data and insights become available. An ablation study would be a valuable next step to systematically assess the significance of each feature, enabling the identification of the most critical components for accurate PFM contraction evaluation. This would allow for refinement of the rating system by removing or adjusting less impactful features. This collaborative approach between clinicians and engineers is crucial for optimizing the overall rating scale, ensuring it meets clinical needs and enhances the reliability and accuracy of PFM contraction assessments.

The implementation of the autonomous maximal PFM contraction performance classifier represents an important advancement in the objective assessment of PFM contraction. Future research should focus on validating the accuracy of the algorithm across different tasks and exploring its potential for integration into clinical practice. The use of objective assessment tools, such as the maximal PFM contraction performance classifier, has the potential to improve the accuracy of PFM contraction assessment and ultimately lead to better assessment and treatment of pelvic floor disorders.

Standardizing the process for evaluating PFM function is crucial since the features evaluated are dependent on the specific task instructions. Therefore, it is necessary to establish standardization across protocols and normalize the different measurement data. In the past, comparing measurements obtained from different devices was challenging due to variations in device design and measurement conditions [16]. However, by standardizing each task, the performance classifier can be generalized across all devices, regardless of the range of PFM force measured. The performance classifier is a promising tool in the standardization of PFM function evaluation across different devices; however, the testing of the performance classifier on different PFM strength measuring devices should be further assessed.

The PFM contraction performance classifier is designed to be agile through accommodating changes in specific task instructions. For example, if a task requires multiple features, the classifier can be adjusted accordingly for rating purposes. This characteristic makes the classifier adaptable to various clinical settings where tasks and instructions may vary.

In future work, the quality assessment system presented in this study is to be integrated into the novel portable PFM dynamometer utilized at Centre de recherche de l’Institut universitaire de gériatrie de Montréal (CRIUGM). Incorporating the quality assessment system into the PFM dynamometer would offer an automated and reliable tool for evaluating PFM function and assessing the efficacy of PFMT, enhancing the PFM contraction assessment and enabling real-time feedback to patients and clinicians.

Nevertheless, this study has some limitations. The data for evaluating different PFM functions are dependent on the specific tasks and cues provided. Each dynamometric task (rest, contraction, push, and cough) was performed according to explicit instructions from the evaluator, leading to variability in how participants executed the tasks. For example, during the maximum contraction task, some participants gradually increased their contraction strength, while others reached maximum contraction rapidly. This variability in individual strategies can result in differences in the contraction profiles, which affects the consistency of the data. Although our PFM contraction detector was trained to recognize and classify a wide range of contraction profiles, the variability introduced by personal responses to task cues remains a limitation. In future studies, more standardized task instructions could help reduce this variability and improve data consistency.

Further, although our system demonstrates high generalizability accuracy, it is important to recognize that its applicability may be influenced by the specific criteria used to define PFM contractions and non-contractions. Additionally, the data used are dependent on specific clinicians’ instructions given for each task. This highlights the need for standardized criteria and protocols. Standardizing how PFM contractions are measured would improve the applicability of the system, making it more consistent across various clinical and research settings.

A final limitation of our study is the use of rest, push, and cough tasks as proxies for non-PFM contraction. We used the tasks at our disposal, which provided indirect information that we hypothesized to represent non-contraction. As these tasks serve as proxies, further studies should aim to test with data recorded directly from incorrect contractions to validate and enhance the accuracy of the quality assessment system.

## 6. Conclusions

In this paper, we presented a novel PFM contraction quality assessment system for the detection and evaluation of PFM contractions in women. The proposed system is composed of a CNN-based PFM contraction detector and an original maximal PFM contraction performance classifier. The CNN model predicts PFM contraction from non-PFM contractions, providing women with feedback on their training. The performance classifier introduces an original algorithm that autonomously evaluates the quality of maximal PFM contraction across different features and gives an overall score. The new quality assessment system is designed to be implemented in PFM function assessment devices, specifically a newly developed portable PFM dynamometer used at CRIUGM. Overall, the proposed system represents a significant advancement in PFM function evaluation and training, as it provides a smart solution for the assessment of PFM contraction. This system has the potential to improve PFM training and rehabilitation, enabling women to better monitor their PFM contraction.

## Figures and Tables

**Table 1:** Summary of dataset for CNN model.

Dataset	Sample Size	Contraction Data	Rest Data	Cough and Push Data
Dataset 1	318	1800	908	1851
Dataset 2	13	33	32	48

**Table 2:** Features extracted.

Definition	Features	Symbols
Force-generating capacity of a muscle [29].	Maximum force; Average contraction force	$\Delta F_{\max}$, $\bar{F_{c}}$
The ability of the PFM to maintain a sustained contraction over a period of time [29].	Contraction time; Area under the curve	$T_{c}$, $AUC_{c}$
The ability of the PFM to work together effectively and efficiently to perform their various functions [29].	Rising slope, Falling slope	$m_{r}$, $m_{f}$
The ability to relax the PFM muscle to its original resting tone following the voluntary contraction [29].	Difference between pre- and post-contraction baseline	$\Delta F_{f-i}$

**Table 3:** CNN model performance results.

Dataset	Accuracy	Sensitivity	Specificity
Dataset 1	96.66%	96.66%	93.75%
Dataset 2	100%	100%	100%

**Table 4:** Digital palpation classification report based on different methods.

Method	Accuracy	Precision	Recall	F1 Score
XGB	90.09%	0.76	0.77	0.76
LR	92.47%	0.83	0.83	0.82
TRFC	92.93%	0.83	0.82	0.81
RFC	95.56%	0.89	0.88	0.88

**Table 5:** Classification report for PFM strength based on digital palpation.

Classes	Precision	Recall	F1 Score	Support
MOS = 1	1.00	0.83	0.90	6
MOS = 2	0.77	0.75	0.76	32
MOS = 3	0.89	1.00	0.94	59
MOS = 4	0.90	0.94	0.92	77
MOS = 5	1.00	0.62	0.76	24
Macro Avg	0.91	0.83	0.86	198
Weighted Avg	0.89	0.88	0.88	198

**Table 6:** Error evaluation of extracted features.

Category	MAE	Standard Deviation of Error	RMSPE
Rising slope, $m_{r}$	0.0018	0.0039	1.138%
Area under the contraction curve, $AUC_{c}$	0.6343	0.8556	0.256%
Global maximum, $\Delta F_{\max}$	0.0700	1.1505	1.65 %
Average force, $F_{c}$	−0.0009	0.0284	0.654%
Muscle relaxation, $\Delta F_{f-i}$	0.0017	0.0043	0.718%

**Table 7:** Comparison with state-of-the-art.

Method	Visual Inspection [16,33]	Catheter Pressure Transducer [34]	Ultrasound [21,35,36]	Pattern-Recognition Algorithm [20]	Accelerometer [21]	AI-CNN Algorithm (This Work)
Technique	Direct observation	Direct observation and sensor	High-frequency sound waves	AI	Sensor	AI
Type of method	Manual evaluation	Manual evaluation	Imaging technology	Machine learning	Rule-based	Machine learning
Data used	Perineum movement	Perineum movement and intravesical pressure values	Sonograms	Exercise technique	PFM contraction force	PFM contraction force
Training requirements	Expert	Expert	Expert	No training needed	No training needed	No training needed
Applicability	Clinical/research	Clinical/research	Clinical/research	No constraints	No constraints	No constraints
Evaluation scheme	Correct, incorrect	Correct, incorrect	Correct, incorrect	Bad (−1) to good (1)	Correct, incorrect	Correct, incorrect
						Features and overall rating scores
Dataset	N/A	N/A	N/A	Sample: 28	Sample: 30	Sample: 331
Accuracy	Operator-dependent	Operator-dependent	Operator-dependent	Not reported	92–100%	96.66–100%
Sensitivity	Operator-dependent	Operator-dependent	Operator-dependent	Not reported	80.3–93.1%	96.66–100%
Specificity	Operator-dependent	Operator-dependent	Operator-dependent	Not reported	90.7–93.1%	93.75–100%
Efficiency	Limited speed	Limited speed	Time consuming	Automated calculation	Automated calculation	Automated calculation
Ease of use	Complex	Highly complex	Complex	Simple	Simple	Simple
Limitations	Subjective, expert need	Subjective, expert need, complex	Subjective, expert need, high cost	Method lacks evaluation	Additional validation testing is required	Wider range of incorrect cases needed

## Data Availability

The datasets presented in this article are not readily available because they contain sensitive personal information and are subject to privacy regulations. Requests to access the datasets should be directed to Chantale Dumoulin at chantal.dumoulin@umontreal.ca.

## Abbreviations

The following abbreviations are used in this manuscript:

UI	Urinary Incontinence
PFM	Pelvic Floor Muscle
AI	Artificial Intelligence
CNN	Convolutional Neural Network
PFMT	Pelvic Floor Muscle Training
MVC	Maximum Voluntary Contraction
RFC	Random Forest Classifier
XGB	Extreme Gradient Boosting
LR	Logistic Regression
MOS	Modified Oxford Scale
ROC	Receiver Operating Characteristic
AUC	Are Under the Curve
CRIUGM	Centre de recherche de l’Institut universitaire de gériatrie de Montréal
